# Determinants of post-campaign measles vaccination coverage in Madagascar: A geographically stratified analysis

**DOI:** 10.1371/journal.pgph.0006390

**Published:** 2026-08-21

**Authors:** Félix Alain, Faly Hariniaina Randrianasolo, Mamy Jean Jacques Razafimahatratra, Holinirina Ramananjanahary, Mbolatiana Raharinivo, Mialy Mathieu Andriambelo, Jean Claude Andrianirinarison, Paubert Tsivahiny, Hanitriniala Pâquerette Sahondranirina, Lethicia Lydia Yasmine, Diana Ratsiambakaina

**Affiliations:** 1 National Institute of Public and Community Health (INSPC), Antananarivo, Madagascar; 2 Faculty of Medicine, University of Mahajanga, Mahajanga, Madagascar; 3 Faculty of Medicine, University of Antananarivo, Antananarivo, Madagascar; 4 Expanded Programme on Immunisation Directorate (EPID), Antananarivo, Madagascar; 5 Tropical Institute of Odonto-Stomatology of Madagascar (IOSTM), Mahajanga, Madagascar; The University of Sydney, AUSTRALIA

## Abstract

Measles remains a significant cause of childhood mortality globally. Despite effective vaccines, the World Health Organisation reported an estimated 10.3 million measles cases worldwide in 2023, a 20% increase from 2022, primarily due to insufficient vaccination coverage. Madagascar faced a major measles epidemic in 2018–2019, prompting vaccination efforts. A convergent parallel mixed-methods design was utilised, wherein quantitative household surveys and semi-structured qualitative interviews were conducted concurrently. Data were integrated during the interpretation phase to triangulate statistical determinants with contextual stakeholder insights regarding the performance of the October 2024 measles vaccination campaign and its determinants across different geographical areas. This retrospective, cross-sectional Post-Measles Campaign Evaluation (PMCE) was conducted from December 2024 to April 2025, following the October 2024 campaign. It involved quantitative household surveys and qualitative interviews in all 23 regions of Madagascar, targeting children aged 9–59 months and their caregivers. A two-stage stratified random sampling approach distinguished large cities, landlocked, and intermediate districts. Multivariate logistic regressions were performed to identify geographical, socio-demographic, economic, and cultural determinants of post-campaign vaccination coverage. The study revealed a national post-campaign vaccination coverage of 75.3% in 2024, a notable 10-point increase from 65.3% in 2022. However, significant geographical disparities persisted (p-value = 0.0233), with lower coverage in large cities (63.2%) compared to landlocked (78.4%) and intermediate (76.2%) districts. Multivariate analysis showed that residing in a large city significantly reduced post-campaign vaccination coverage (OR adjusted = 0.25; p = 0.010). Conversely, male child gender (OR adjusted = 1.33; p = 0.040), birth in a health facility (OR adjusted = 1.38; p = 0.046), possession of a birth certificate (OR adjusted = 2.30; p < 0.001), having a Christian caregiver (OR adjusted = 1.92; p < 0.001), or a housewife caregiver (OR adjusted = 1.97; p = 0.015) were associated significantly higher post-campaign vaccination coverage. The identified statistical determinants, notably the lower coverage among urban residents (ORa = 0.25), were contextualised using qualitative data, which highlighted that ‘urban vaccine hesitancy’ is exacerbated by metropolitan misinformation and the misconception that previously vaccinated children did not require campaign doses. While Madagascar's post-campaign measles vaccination coverage shows encouraging progress, significant geographical and socio-demographic disparities underscore the need for tailored strategies. Addressing urban-specific challenges, strengthening communication, improving logistics, and leveraging community engagement are crucial for achieving equitable immunisation coverage and advancing towards measles elimination targets.

## Introduction

Measles remains a leading cause of childhood mortality worldwide, despite the availability of an effective and safe vaccine [1]. In 2023, the World Health Organisation (WHO) and the Centres for Disease Control and Prevention (CDC) estimated that there will be 10.3 million measles cases worldwide, marking a 20% increase from 2022, mainly due to insufficient vaccination coverage [2,3]. The global measles elimination initiative aims to reduce the incidence of this disease [4–6].

Madagascar is an island nation with a health system structured in four levels (central, regional, district, and community). The country’s diverse geography, significant rural enclavement, and areas of insecurity represent major limiting factors for national healthcare coverage and vaccination access. In Madagascar, measles is a disease prone to deadly epidemics, particularly affecting young children, which justifies a national elimination programme [7]. Madagascar's Expanded Programme on Immunisation (EPI), established in 1976, has implemented routine systematic vaccination strategies and periodic campaigns to strengthen childhood immunization [8,9]. To supplement routine services, follow-up campaigns have traditionally been implemented every three years. However, despite these efforts, a decline in surveillance performance between 2018 and 2020, combined with low herd immunity, contributed to a massive resurgence of the disease. Madagascar faced a major outbreak starting in September 2018, which affected several major cities and spread to all 22 regions [7–9]. This epidemic resulted in 244,650 notified cases and 1,080 deaths (0.44% lethality). The country responded to this emergency situation with a three-phase response vaccination campaign organised in February 2019, May 2022 and October 2024, targeting children aged 9 months to 59 months [7,8]. The current Post-Measles Campaign Evaluation (PMCE) revealed that the October 2024 campaign achieved a national vaccination coverage of 75.3% [8].

Vaccination coverage estimates show persistent challenges. According to WHO/UNICEF estimates, measles vaccine (MVC) coverage declined from 81% in 2007 to 59% in 2016 and then to 68% in 2018 [8]. The 2021 national coverage survey revealed MVC coverage of 55% [10]. The 2022 Post-Measles Campaign Evaluation (PMCE), meanwhile, found crude vaccination coverage of 65.3% and card-documented coverage of 56.3% [10]. Suboptimisation of vaccination coverage can lead to significant epidemics [9]. Factors contributing to these challenges include insufficient coverage, late implementation of mass vaccination campaigns, limited financial resources, high population mobility, and high disease transmission rates [7]. In addition, insecurity and geographical inaccessibility are major obstacles to accessing medical care and vaccination in Madagascar [10].

In line with its prevention policy and the need to strengthen evidence-based interventions, Madagascar has scheduled measles vaccination follow-up campaigns every three years [10]. To this end, a follow-up campaign was recently implemented in October 2024 across the entire country, targeting more than four million children aged 9–59 months.

In order to evaluate the effectiveness of this campaign and to measure post-campaign vaccination coverage, a Post-Measles Campaign Evaluation (PMCE) was conducted by the National Institute of Public and Community Health (INSPC). This approach is in line with WHO guidelines which recommend an independent survey within one month of the end of the campaign to validate administrative coverage and process quality [11,12]. Generally, this study aims to analyse the determining factors of post-campaign measles vaccination coverage in Madagascar using a stratified approach by geographical areas. More specifically, this study aims to determine post-campaign measles vaccination coverage among children aged 9–59 months in Madagascar at the national level and according to different geographical strata (large cities, landlocked districts, to intermediate districts); identify the sociodemographic, economic and cultural determinants associated with the post-campaign vaccination coverage of children aged 9–59 months, in particular according to the residential environment and the geographical stratum and to identify the main sources of information for parents regarding measles vaccination.

## Materials and methods

This research was part of the Post-Measles Campaign Evaluation (PMCE), a retrospective and cross-sectional survey conducted to assess the impact of the national measles vaccination campaign held in October 2024. A convergent parallel mixed-methods design was utilised, wherein quantitative household surveys and semi-structured qualitative interviews were conducted concurrently. Data were integrated during the interpretation phase to triangulate statistical determinants with contextual stakeholder insights to provide a comprehensive evaluation across all 23 regions of Madagascar.

The recruitment period, which refers to the specific timeframe when participants were formally enrolled into the study, began on 21/12/2024 and concluded on 14/02/2025. Recruitment and field data collection were carried out in two distinct phases: an initial phase in the Analamanga region from 21/12/2024 to 23/12/2024 and a second phase covering the remaining regions from 25/01/2025 to 14/02/2025. While these dates define the formal enrolment of participants, the broader study period, encompassing protocol development, training, and final data analysis, extended from September 2024 to April 2025.

### Ethics statement

Ethical approval was granted by Ethical Evaluation Committee for Biomedical Research (Comité d’Évaluation Éthique pour la Recherche biomédicale - CEER) of the National Institute of Public and Community Health (Institut National de Santé Publique et Communautaire - INSPC) (Ref: 021/2024-CEERINSPC/IRB). Oral informed consent was obtained for quantitative interviews, as securing written consent was not feasible in these large-scale, low-literacy field settings, while signed written informed consent was obtained for qualitative interviews; the use of oral consent was specifically approved by the CEER.

### Type of study, period and target population

This is a retrospective, cross-sectional post-campaign vaccination coverage survey conducted on a random sample of children aged 9–59 months. The study covered all 23 regions of Madagascar. The target populations included children aged 9–59 months at the time of the campaign and their household caregivers. The vaccination campaign period was October 2024.

Inclusion criteria for children were being between 9 and 59 months old at the time of the campaign and having slept in the household the night before the survey. Vaccination status was determined based on a combination of documented evidence from vaccination cards (seen by surveyors) and caregiver recall for children without cards.

The parameters studied included: characteristics of caregivers in the household, characteristics of eligible children surveyed; information on the campaign (exposure to information, sources of information, knowledge of the disease); post-campaign vaccination coverage; reasons for non-vaccination; incidence of Adverse Events Following Immunisation (AEFI) and use of care; routine vaccination status of children and social and behavioural factors influencing vaccination.

### Sampling

A two-stage stratified cluster random sampling technique was employed, which constitutes a multistage sampling approach. In this design, enumeration areas (EAs) were selected as primary units in the first stage, and households were selected in the second stage. Districts were classified into three geographic strata to reduce variance and increase the precision of the estimates: (1) large city districts based on high urban density; (2) landlocked districts characterised by limited road access and significant geographic barriers; and (3) intermediate districts. The sampling frame was the 2017 census mapping of INSTAT (National Institute of Statistics), also used for the PMCE 2022 and the EDSMD-V 2020. This frame was prepared according to the three strata, excluding insecurity zones before the selection of the enumeration areas, which constituted the primary sampling units.

The sample size was calculated by stratum. Initially, the target was 2,600 households divided into 104 clusters, targeting approximately 1,818 children aged 9–59 months. The expected post-campaign coverage for zero-dose children was 85% for the calculation: (i) stratum 1 (large cities): 36 clusters, 900 households, 607 children expected; (ii) stratum 2 (landlocked districts): 34 clusters, 850 households, 599 children expected; and (iii) stratum 3 (intermediate districts): 34 clusters, 850 households, 612 children expected.

The sampling process took place in several stages:

1. **Selection of ZDs:** Carried out from the INSTAT database, with a draw proportional to the size of the ZDs for each stratum.
2. **Segmentation and segment selection:** EAs that were too large (> 60 households or spanning several localities) were divided into segments, and one segment was selected for each cluster.
3. **Recognition, delimitation of clusters and enumeration of households:** In the field, the survey teams confirmed their location with the controllers and counted all the households in the cluster.
4. **Household selection:** A detailed guide was used by the supervisor to select the 25 households to be visited in each cluster.
5. **Child selection:** All children aged 9–59 months from the selected households were included, and their data were collected.

A total of 101 clusters out of the 104 selected were effectively covered (completion rate of 97.1%), and 2,437 households were surveyed with an acceptance rate of 99.3%.

### Data collection

Data collection combined quantitative and qualitative approaches. Quantitative household surveys were conducted using tablets via the Kobo Collect application. Data included information on households, the residential environment, and the characteristics of children and caregivers. To ensure the scientific rigour and credibility of qualitative findings and minimise bias, qualitative data were gathered through semi-structured interviews with 227 health system stakeholders (61 regional and district health officials and 166 community leaders and agents) selected through purposive sampling across different levels of the health system. Specific questionnaires were developed for these interviews. Data collection took place in two phases: a first phase in the Analamanga region (December 21–23, 2024) and a second phase in the other regions (January 25 - February 14, 2025).

### Data processing and analysis

Data collected via Kobo Collect was downloaded and cleaned using Excel and SPSS. STATA (version 17.0) was used for all main statistical analyses, including multivariate modelling and adjustments for the complex survey design. We used Zotero to manage the references.

1. **Weighting of observations:** Household weighting was applied for each household, taking into account the probability of inclusion of EAs and the probability of selection of households, as well as an a posteriori adjustment for the non-response rate.
2. **Univariate and bivariate statistical analysis:** Descriptive and comparative analyses were carried out on the main indicators and variables (gender and ages of children, urban/rural environment, stratum, socio-economic and cultural characteristics of household heads and caregivers). A significance threshold of 0.05 was set, with 95% confidence intervals.
3. **Multivariate analysis:**

Multivariate logistic regression models were constructed using survey-set (svy) commands in STATA to account for the complex survey design, including clustering, stratification, and sampling weights. This approach was utilised to identify the determinants of exposure to campaign information, post-campaign vaccination coverage, and routine childhood vaccination status while ensuring the correct estimation of standard errors and 95% confidence intervals. These models calculated adjusted odds ratios (OR_a) and took into account geographic, sociodemographic, economic, and cultural factors.

1. **WHO Vaccination Coverage Quality Indicators (VCQI) model:** This tool was used to describe the sample, determine national coverage by subgroups (gender, age, area of residence, education level), determine the proportion of children who received their first dose of VAR and identify low coverage clusters.
2. **Qualitative Analysis:** To ensure scientific rigour and replicability, qualitative data underwent thematic content analysis adhering to the Framework Method (Ritchie & Spencer) [13], comprising: (1) transcription and familiarisation (including translation of interviews into a format for analysis); (2) systematic coding to organise data units systematically; (3) analytical framework development and application to group recurring codes into categories; and (4) thematic synthesis to identify constants and relationships between participants. To further enhance validity, data were triangulated across diverse stakeholder groups. Dedicated analysis workshops were conducted in February and March 2025 to complete transcriptions and ensure the quality of the cross-sectional analysis. The qualitative investigation and reporting followed the Consolidated Criteria for Reporting Qualitative Research (COREQ) [14]. The complete 32-item checklist is included as S1 File.

## Results

The study included 1,294 eligible children (Large cities: n = 363; Landlocked: n = 410; Intermediate: n = 521). The national post-campaign vaccination coverage was 75.3%. This represents a significant 10-point increase compared to the 65.3% coverage recorded in 2022. Analysis of post-campaign vaccination coverage identified significant disparities across geographic strata (p-value = 0.0233). Coverage was notably lower in the large cities stratum (63.2%) compared to intermediate districts (76.2%) and landlocked districts (78.4%). This observed trend of lower urban coverage is consistent with findings from the 2022 PMCE. Nationally, vaccination coverage did not significantly differ between rural (75.9%) and the broader urban (70.5%) areas (p-value = 0.4300).

### Post-campaign vaccination coverage and geographic disparities

**Fig 1:**
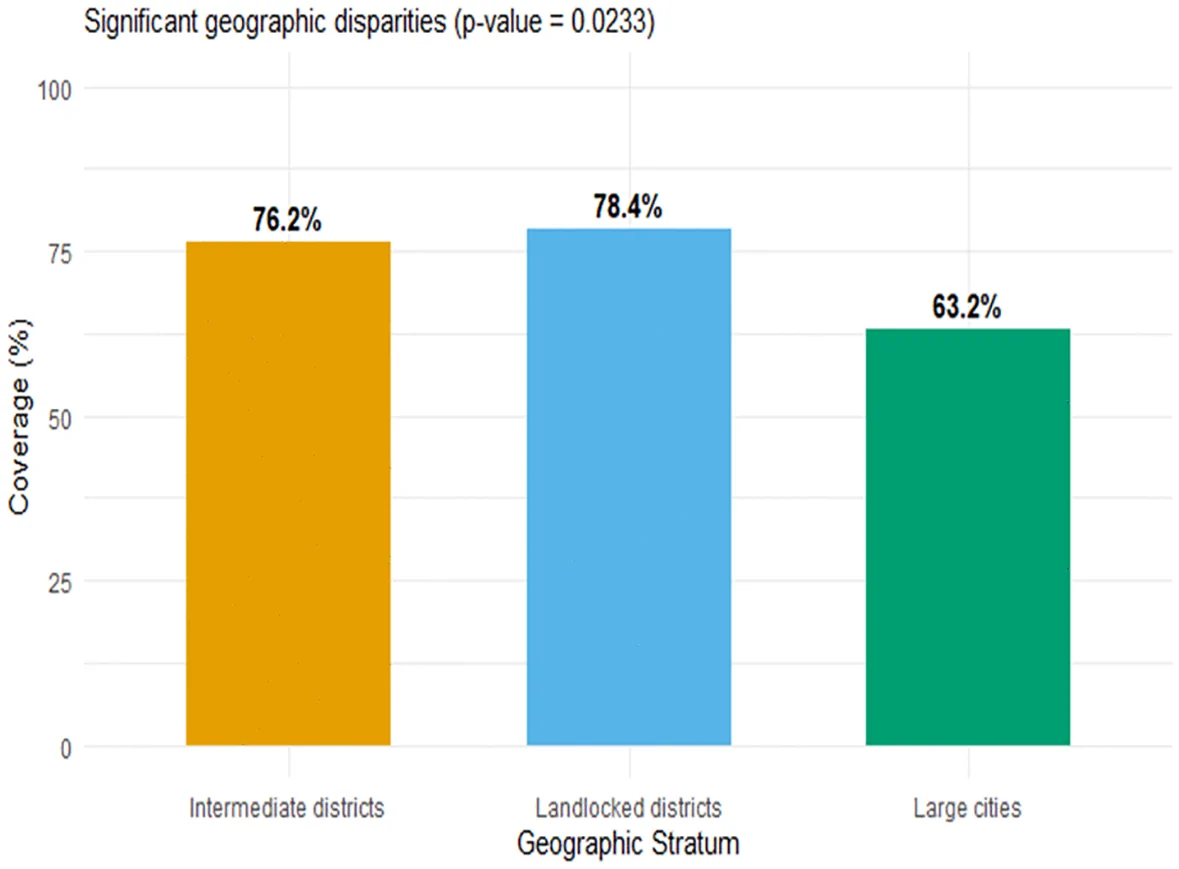
Post-campaign measles vaccination coverage by stratum (2024).

The national and stratified post-campaign measles vaccination coverage is detailed in Table 1 and Fig 1. While overall national coverage reached 75.3% (95% CI: 69.3, 80.5), significant regional variations persisted. Landlocked districts achieved the highest coverage at 78.4% (95% CI: 70.1, 84.9), followed by intermediate districts at 76.2% (95% CI: 68.9, 82.3), whereas large cities recorded the lowest coverage at 63.2% (95% CI: 55.7, 70.0).

**Table 1:** Post-campaign measles vaccination coverage in Madagascar in 2024 by stratum and residential area.

Features	Vaccinated during the AVS (%)	95% CI (%)	P-value
MADAGASCAR (n = 1,294)	75.3	[69.3, 80.5]	
**Stratum**			**0.0233**
Big cities (n=363)	63.2	[55.7, 70.0]	
Landlocked districts(n=410)	78.4	[70.1, 84.9]	
Intermediate districts (n=521)	76.2	[68.9, 82.3]	
**Place of residence**			**0.4300**
Rural	75.9	[69.2, 81.6]	
Urban	70.5	[55.4, 82.2]	

A notable challenge was the low rate of vaccination card presentation; only 33.3% of vaccinated children had their vaccination cards seen by enumerators. This proportion varied by stratum, from 24.6% in large cities to 38% in landlocked districts, highlighting potential limitations in vaccination documentation.

## Analysis of the determinants of post-campaign vaccination coverage (Multivariate analysis)

### Campaign information exposure and reasons for non-vaccination

Among the caregivers interviewed, 70.5% reported being informed before the campaign started. Community mobilisers (52%) were the most frequently cited information source nationally, followed by radio (28%), health agents (22%), and neighbours/friends (21%). Community agents/volunteers were consistently the primary source across all strata, accounting for 49% of all reported information sources. Exposure to campaign messages through health centres varied significantly across strata (p-value = 0.001); households in landlocked districts were most exposed (34.1%), followed by intermediate districts (22.6%), and least exposed in large cities (18.9%).

For reasons of non-vaccination, insufficient information (43%) was the leading national reason, followed by lack of opportunity (28%), obstacles (18%), and lack of motivation (11%). In large cities, insufficient information (62%) was particularly prominent, often linked to a lack of exposure to campaign messages and the belief that previously vaccinated children were not concerned. In landlocked districts, however, lack of opportunity (48%) was the main reason.

### Adverse Events Following Immunisation (AEFI) incidence and management

The overall national incidence of Adverse Events Following Immunisation (AEFI) reported by parents was 4.6%. AEFI incidence was higher in large cities (7.9%) and urban settings (5.9%), and notably higher among girls than boys. The majority of respondents (66.1%) perceived these events as “not serious,” while 28.2% considered them “serious,” and only 0.7% “very serious.” Despite the occurrence of AEFIs, 94.7% of parents expressed willingness to continue using vaccination services. Approximately six out of ten AEFI cases received advice or treatment, primarily from health centres (49.6%) and community agents (25.8%). Qualitative findings further confirmed that no severe AEFI cases were recorded during the campaign, with only minor symptoms like slight fevers observed by health officials.

### Routine immunisation status and social norms

Analysis of routine immunisation status among children aged 9–59 months revealed that 16.7% were zero-dose, 26.2% were incompletely vaccinated, and 57.3% were completely vaccinated at the national level. This status varied significantly by geographic stratum (p-value <0.001). Large cities showed the best routine vaccination performance (5.1% zero-dose, 19.1% incomplete, 76.0% complete), followed by landlocked districts (8.1% zero-dose, 22.6% incomplete, 69.5% complete), and intermediate districts (18.5% zero-dose, 27.2% incomplete, 54.5% complete). Urban areas also exhibited better routine vaccination rates (4.0% zero-dose, 18.1% incomplete, 78.0% complete) compared to rural areas (18.3% zero-dose, 27.3% incomplete, 54.6% complete).

Regarding knowledge and perceptions:

- Knowledge of specific vaccines was generally higher in urban areas, with BCG, measles (VAR), and polio (VPO) being the most recognised antigens.
- The majority of caregivers (79.2% nationally) considered vaccines “very important” for their child's health. Similarly, 75.7% believed vaccines to be “very safe,” and 76% expressed “great confidence” in health agents.
- Influence of leaders: 26% of caregivers reported religious leaders encouraging vaccination, while 60% reported that they did not receive such encouragement. The remaining 15% of respondents were unsure of their religious leader's position. In contrast, 69% of caregivers reported local leaders encouraging vaccination.
- Access: Nearly 95% of caregivers knew where to vaccinate their children. However, 8.8% reported being turned away from vaccination services.

### Multivariate analysis of post-campaign vaccination coverage determinants

Multivariate logistic regression models identified key determinants of post-campaign vaccination coverage and routine vaccination status at the national level and by stratum. The results of this analysis are summarised in Table 2.

**Table 2:** Summary of the results of the logistic models of post-campaign vaccination coverage at the national level and by stratum.

Factors	Madagascar			Big cities			Landlocked districts			Intermediate districts		
	OR_a	95% CI	p-value	OR_a	95% CI	p-value	OR_a	95% CI	p-value	OR_a	95% CI	p-value
**Stratum**												
Intermediate districts	1*											
Big cities	0.25	[0.09, 0.71]	0.010	N/A			N/A			N/A		
Landlocked districts	1.48	[0.81, 2.70]	0.203	N/A			N/A			N/A		
**Place of residence**												
Urban	1*											
Rural	0.98	[0.32, 3.03]	0.972	N/A			13.98	[1.93, 101.2]	0.009		[N/A]	
**Gender of the child**												
Girl	1*											
Boy	1.33	[1.01, 1.74]	0.040	0.75	[0.33, 1.69]	0.487	1.06	[0.29, 3.82]	0.929	1.44	[1.06, 1.95]	0.018
**Birth in health facility**												
No	1*											
Yes	1.38	[1.01, 1.89]	0.046	1.25	[0.47, 3.33]	0.655	1.94	[0.50, 7.48]	0.336	1.31	[0.91, 1.88]	0.136
**A birth certificate**												
No	1*											
Yes	2.30	[1.63, 3.25]	<0.001	1.60	[0.17, 15.2]	0.682	2.27	[0.54, 9.54]	0.263	2.23	[1.49, 3.34]	<0.001
**Religion of person in charge**												
Other/No religion	1*											
Christian	1.92	[1.37, 2.68]	<0.001	1.90	[0.46, 7.89]	0.377	0.44	[0.10, 1.93]	0.276	2.06	[1.43, 2.96]	<0.001
**Respondent's profession**												
Other/NP	1*											
Housewife	1.97	[1.14, 3.41]	0.015	0.84	[0.22, 3.17]	0.797	1.57	[0.15, 16.2]	0.705	2.24	[1.11, 4.51]	0.024

1*: reference

**Fig 2:**
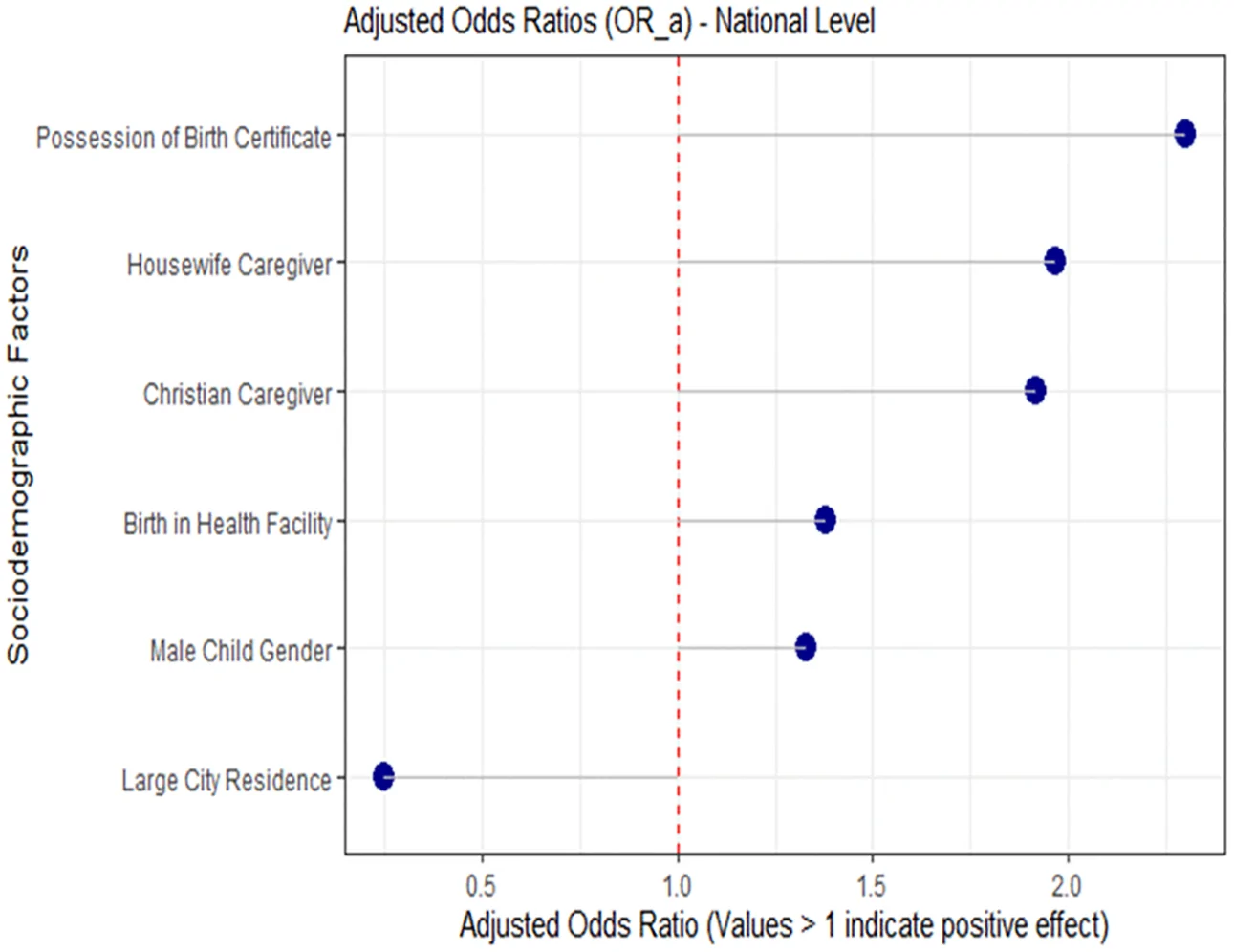
Determinants of post-campaign vaccination coverage.

At the national level (See Fig 2), for post-campaign vaccination coverage:

- Residing in a large city significantly reduced a child's post-campaign vaccination coverage by approximately four times (Adjusted Odds Ratio (OR_a) = 0.25; p-value = 0.010).
- A child's male gender increased post-campaign vaccination coverage by 1.33 times (OR_a = 1.33; p-value = 0.040).
- Birth in a health facility increased post-campaign vaccination coverage by 1.38 times (OR_a = 1.38; p-value = 0.046).
- Possession of a birth certificate was strongly associated significantly higher post-campaign vaccination coverage, by 2.3 times (OR_a = 2.30; p-value < 0.001).
- A Christian caregiver increased post-campaign vaccination coverageby 1.92 times (OR_a = 1.92; p-value < 0.001).
- A caregiver who is a housewife increased post-campaign vaccination coverageby 1.97 times (OR_a = 1.97; p-value = 0.015).

For intermediate districts, for post-campaign vaccination coverage:

- A child's male gender increased post-campaign vaccination coverage by 1.44 times (OR_a = 1.44; p-value = 0.018).
- Possession of a birth certificate increased post-campaign vaccination coverage by 2.23 times (OR_a = 2.23; p-value < 0.001).
- A Christian caregiver increased post-campaign vaccination coverage by 2.06 times (OR_a = 2.06; p-value < 0.001).
- A caregiver who is a housewife increased post-campaign vaccination coverage by 2.24 times (OR_a = 2.24; p-value = 0.024).

For zero-dose routine vaccination at the national level, residing in landlocked districts reduced the risk by 0.431 times (OR_a = 0.431, p-value = 0.047), and having a sedentary caregiver reduced the risk by 2 times (OR_a = 0.50, p-value = 0.047). For incomplete routine vaccination nationally, having a zero-dose status increased the risk of incomplete vaccination by 2.77 times (OR_a = 2.77, p-value = 0.002), while a sedentary caregiver reduced this risk by approximately 2 times (OR_a = 0.51, p-value = 0.04).

### Qualitative study findings

Semistructured interviews with 227 health system stakeholders provided deeper insights into campaign implementation and community perceptions.

### Reasons for vaccination refusal

The primary drivers of vaccination opposition in Madagascar were found to be religious and socio-cultural beliefs, concerns about potential side effects, and lower levels of education. Hostility towards vaccinators and social prohibitions related to injections were also significant barriers. A successful strategy to address distrust and mitigate the specific coverage barriers identified in large cities was to recruit actors from within the local community to join vaccination teams.

### Campaign coordination and planning

Overall, coordination was generally effective at central and regional levels, with notable involvement of stakeholders. Micro-planning involved bottom-up approaches, and a rapid response sub-committee addressed field challenges. However, a key difficulty was the lack of coherence between the validated microplan and the actual needs of Basic Health Centres (CSBs).

### Logistics and supply chain

Significant logistical challenges included the non-receipt of vaccination cards by 16% of vaccinated children during the campaign and the low presentation rate of only 33% of documentation to enumerators during the survey. Late arrival of essential inputs, such as vaccines and markers, also affected some districts.

### Communication and mobilisation

Diverse communication channels were employed, including community mobilisers, radio spots, and posters, alongside direct engagement by health agents. Social media and the internet played a negligible role.

### Training, supervision, and data management

While there were noted gaps in training for vaccination managers (40% of District Public Health Services (SDSPs) and 93% of CSBs reported not being trained), all 9 surveyed regions established supervision plans, and 76% of planned supervision visits were successfully conducted. Supervision teams actively contributed to managing cases of vaccine hesitancy. Data collection utilised Computer-Assisted Personal Interviewing (CAPI) via Kobo Collect, ensuring real-time data flow. The survey achieved a high cluster coverage rate (97.1%) and a household response rate of 99.3%. Independent monitoring results were used proactively to organise additional vaccination sessions in areas with low coverage.

### AEFI management

Consistent with quantitative findings, qualitative data indicated no severe AEFI cases were recorded during the campaign, with only minor symptoms like slight fevers observed by health officials. However, direct district and regional involvement in AEFI management was limited as no severe cases were reported. Community agents sometimes hesitated to report AEFIs due to concerns about linking them directly to vaccination.

### Waste management

Generally, almost all health agents contributed to the management and destruction of waste.

## Discussion

The post-campaign measles vaccination coverage of 75.3% observed in Madagascar in 2024 reflects encouraging progress, marking a 10-point increase compared to the 2022 campaign's 65.3% coverage [10]. This demonstrates the impactful efforts of the health system and partners. However, this coverage still falls short of the recommended 95% threshold necessary for widespread immunity and measles elimination [15]. Our findings highlight critical geographical disparities and socio-demographic determinants that influence vaccination uptake, offering valuable insights for future immunisation strategies in Madagascar.

### National coverage and geographic disparities

Our study's finding of a 75.3% national post-campaign vaccination coverage in 2024 is a positive step, surpassing previous administrative coverages in Madagascar, which were around 60% from 2014-2018, contributing to outbreaks [7]. However, significant geographic inequities persist, with notably lower coverage in large cities (63.2%) compared to landlocked (78.4%) and intermediate districts (76.2%). This mirrors a trend observed in the 2022 PMCE [10] and aligns with similar challenges documented in other low- and middle-income countries (LMICs) [15]. For instance, a geospatial analysis in Nigeria revealed that while campaigns generally achieved higher and more homogeneous coverage than routine immunisation (RI), significant geographic inequities remained at granular levels, with certain “coldspots” consistently being missed by both strategies [16]. Urban areas, often assumed to have better access, frequently present unique challenges, including high population mobility, diverse socio-economic strata, and potential for greater vaccine hesitancy, which can lead to lower coverage compared to more stable rural or intermediate settings [17].

### Determinants of post-campaign vaccination coverage

Our multivariate analysis identified several key determinants of post-campaign vaccination coverage (Table 2). Residing in a large city was a strong negative determinant, reducing a child’s post-campaign vaccination coverage by approximately four times (OR_a = 0.25; p = 0.010). This is a consistent challenge across Africa, where urban settings can present “urban immunisation challenges” due to complex population dynamics and diverse belief systems [17]. This finding underscores the need for “tailored approaches” to address vaccine hesitancy and improve access in metropolitan areas, as advocated by WHO [18,19]. Conversely, a child's male gender (OR_a = 1.33; p = 0.040) was associated with higher post-campaign vaccination coverage. The possession of a birth certificate (OR_a = 2.30; p < 0.001) was a significant positive determinant, consistent with studies in Kenya demonstrating a link between birth registration and vaccination uptake [20]. This likely reflects better engagement with formal health systems and higher socio-economic status. Birth in a health facility (OR_a = 1.38; p = 0.046) was also positively associated with vaccination, indicating that early contact with the health system facilitates subsequent immunisation adherence. This is also linked to the observation that health system strengthening (HSS) efforts can improve timely vaccination and reduce economic inequalities by increasing routine immunisations, although these benefits may disproportionately favour those closer to health facilities [9]. A Christian caregiver (OR_a = 1.92; p < 0.001) and a caregiver being a housewife (OR_a = 1.97; p = 0.015 nationally) were also positive determinants. Religious beliefs can be a complex factor, sometimes fostering trust and sometimes leading to vaccine refusal [18,21]. The role of housewives may reflect greater availability for community engagement or a closer connection to health information networks.

### Communication, misinformation, and refusal

Our study highlighted that 70.5% of caregivers were informed before the campaign, with community mobilisers (52%) being the most frequently cited source. This underscores the critical role of interpersonal communication and community health workers in successful vaccination campaigns [22]. However, insufficient information (43% nationally, 62% in large cities) was a primary reason for non-vaccination, indicating persistent gaps in communication outreach, particularly in urban settings [10]. Qualitative data further revealed that refusal stems from religious and socio-cultural beliefs, fear of adverse effects, and lower education levels, alongside “hostility towards vaccinators and social prohibitions related to injections.” These barriers are common across African contexts, where misinformation and distrust can impede campaign success [17,21]. To enhance transparency, qualitative findings from 227 stakeholders were synthesised to contextualise the statistical determinants; for instance, the success of community-led recruitment in addressing identified ‘hostility towards vaccinators’ provides a context-specific strategy to mitigate the barriers observed in large cities.

### Routine immunisation integration and “Zero-Dose” children

The campaign provided a crucial opportunity to reach children missed by routine immunisation (RI). Nationally, 16.7% of children were zero-dose (having received no basic vaccines). Our finding that residing in landlocked districts reduced the risk of a child being zero-dose (OR_a = 0.431; p = 0.047) suggests better, or more consistent, access to RI services in these areas compared to large cities, where systemic challenges might lead to more missed children. Despite supplementary immunisation activities (SIAs) regularly boosting coverage, the recurrence of measles outbreaks in many areas emphasises the need for improved RI for long-term measles control [23]. This underscores that SIAs should not be viewed as substitutes for robust RI but rather as complementary strategies [16]. Efforts to strengthen RI programmes, including increasing the availability and retention of vaccination records, are critical for achieving measles elimination goals [9]. Low RI coverage has historically been a challenge in Madagascar, contributing to outbreaks [7].

### Adverse Events Following Immunisation (AEFI) Management

The reported AEFI incidence was low (4.6% nationally), with the majority perceived as “not serious” and a high willingness of parents (94.7%) to continue using vaccination services despite their occurrence. Importantly, no severe AEFI cases were recorded according to both quantitative and qualitative data. This demonstrates a relatively high level of community trust in the vaccination programme and the health system's ability to manage minor side effects. However, qualitative insights indicated that AEFI notification might not be optimal at all levels, and more extensive information to parents about potential AEFIs could further reduce fears and improve confidence [24,25].

### Programmatic implications

Our findings underscore the urgent need for stratified and context-specific strategies in Madagascar, especially in large cities where coverage is lowest and specific barriers, such as misinformation and the belief that previously vaccinated children are not concerned, are more prevalent. Interventions could include adapting mobile outreach and ensuring robust communication campaigns that address common misconceptions and actively involve local and religious leaders [19]. The significant logistical challenges, such as the non-receipt of vaccination cards by 16% of vaccinated children during the campaign and the low presentation rate of only 33% of documentation to enumerators during the survey, demand improved supply chain management and communication efforts to emphasise the importance of retaining vaccination records [10]. Enhancing training for health personnel, particularly at the CSB level, and ensuring timely financial resource disbursement are also critical to optimise campaign implementation and overcome persistent operational weaknesses.

### Study strengths and limitations

This study's strengths include its stratified design and national representativeness across all 23 regions of Madagascar, ensuring robust and generalisable quantitative estimates. The combination of quantitative household surveys and qualitative interviews provided a comprehensive understanding of both the determinants of coverage and the underlying perceptions and operational challenges. This mixed-methods approach offers valuable insights that purely quantitative studies might miss [17,26].

However, the study is subject to several limitations. First, as a cross-sectional design, it does not allow for the inference of direct causality between determinants and coverage. Second, potential recall bias from caregiver self-reporting of vaccination history, particularly given the low vaccination card presentation rate (33.3%), may have affected the accuracy of coverage estimates [15,27]. These are common challenges in similar evaluations in LMICs [17,26]. Third, the exclusion of three enumeration areas (EAs) due to insecurity and access difficulties represents a limitation, although post-stratified adjustments were applied to correct the weights. Finally, the delay of over six weeks between the October 2024 campaign and data collection (concluding in April 2025 in some areas) could have introduced memory bias regarding specific campaign details.

### Unanswered questions and future research

Further research is needed to investigate the specific “systemic challenges” and mistrust that contribute to homogeneous low coverage in large cities, as suggested by our findings. Understanding the nuanced reasons behind vaccine refusal linked to religious and socio-cultural beliefs requires in-depth ethnographic studies. Longitudinal studies could help establish causal links between identified determinants and vaccination uptake. Additionally, detailed cost-effectiveness analyses of tailored interventions in urban and hard-to-reach areas could guide resource allocation for future campaigns. Finally, improving the availability and retention of vaccination records remains a critical area for future efforts, as this would enhance the accuracy of coverage surveys and facilitate routine immunisation monitoring.

## Conclusion

The study concludes by emphasising the crucial importance of socio-demographic, economic, and cultural factors, alongside geographical disparities, for improving future vaccination strategies in Madagascar. While national vaccination coverage showed an encouraging increase compared to previous years, it still falls short of the recommended level for achieving widespread immunity against measles.

Significant geographical differences were observed, with large cities displaying lower vaccination coverage compared to intermediate and landlocked districts. This pattern suggests a particular challenge often referred to as “urban vaccine hesitancy.”

At the national level, key determinants influencing post-campaign vaccination coverage included the child's male gender, birth in a health facility, possession of a birth certificate, having a Christian caregiver, and a caregiver identified as a housewife. Conversely, residing in a large city was associated with a reduced probability of being vaccinated during the campaign. Interestingly, within large cities, no specific distinguishing factors were identified, suggesting a homogeneous, yet generally low, adherence. In landlocked districts, rural residence emerged as a strong positive factor, possibly due to robust community mobilisation efforts. Intermediate districts exhibited determinants similar to those found nationally.

These findings highlight the urgent need for tailored, urban-specific strategies to effectively address vaccine hesitancy in metropolitan areas. Additionally, strengthening civil registration, promoting deliveries in health facilities, and fostering partnerships with religious organisations and women's networks are crucial interventions for enhancing vaccination success and building trust across Madagascar.

## Supporting information

S1 File COREQ: 32-Item Checklist for Qualitative Research. The complete 32-item checklist used for reporting qualitative findings, adhering to the Consolidated Criteria for Reporting Qualitative Research (COREQ).
(DOCX)

## Abbreviations

AEFI: Adverse Events Following Immunisation
CAPI: Computer-Assisted Personal Interviewing
CDC: Centres for Disease Control and Prevention
CSBs: Basic Health Centres
DPEV: Directorate of the Expanded Programme on Immunisation
EPI: Expanded Programme on Immunisation
INSPC: National Institute of Public and Community Health
INSTAT: National Institute of Statistics
LMICs: Low- and middle-income countries
MVC: Measles vaccine
PMCE: Post-Measles Campaign Evaluation
SDSPs: District Public Health Services
SIAs: Supplementary immunisation activities
VAR: Measles Vaccine
