# Supplementary material for: Determinants of post-campaign measles vaccination coverage in Madagascar: A geographically stratified analysis
Source: PLOS Glob Public Health. 2026 Aug 21;6(8):e0006390. doi: 10.1371/journal.pgph.0006390 (PMC13496406; doi:10.1371/journal.pgph.0006390)
Supplement: Supplementary file 1 [file pgph.0006390.s003.pdf]

Dr. Félix ALAIN,
Corresponding Author, Antananarivo,
Madagascar
E-mail: alainfelixmed@gmail.com

Antananarivo, May 24, 2026

To: Editorial Office, PLOS Global Public Health

<u>**Subject:**</u> **Resubmission of Revised Manuscript (ID: PGPH-D-26-01253)**

Title: Determinants of post-campaign measles vaccination coverage in Madagascar: A geographically stratified analysis

Dear Editor,

We would like to thank the editors and the reviewers for their constructive feedback on our manuscript. We have carefully addressed all the requested revisions to improve the clarity, context, and methodological transparency of our study.
As requested, we are submitting the following documents:

1. This point-by-point response document;
2. A marked-up copy of the manuscript with track changes;
3. A "Clean Copy" of the revised manuscript;
4. A completed COREQ checklist (as Supporting Information).

We believe these changes have significantly strengthened the paper and look forward to your decision.

Sincerely,

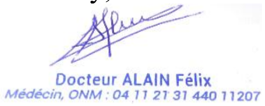

Table 1: Addressing Comments from Reviewer 1

N°	Examiner comment (Full Quote)	Original sentence/phrase (in the manuscript)	New/improved sentence/phrase	Justification / rationale
**1**	"Phrasing can be tightened for structure and clarity I would suggest the following: Determinants of post-	"Analysis of the determinants of post-campaign measles vaccination coverage in	"Determinants of post-campaign measles vaccination coverage in Madagascar: A geographically stratified analysis"	Revised the title to be more concise and strictly follow the

	campaign measles vaccination coverage in Madagascar; A geographically stratified analysis."	Madagascar: A stratified approach by geographical areas"		reviewer's recommendation to enhance structural clarity
**2**	"Discuss the geography of Madagascar... what has been done previously in immunization... to mirror the study much better."	"In Madagascar, measles is a disease prone to deadly epidemics, particularly affecting young children..."	"Madagascar is an island nation characterized by diverse geography and varying levels of health service accessibility... Previous follow-up campaigns were implemented every three years until 2016, but surveillance performance declined between 2018 and 2020, contributing to a massive resurgence..."	Added detailed geographic and historical context to strengthen the justification for the study's stratified approach and provide essential background on the 2018–2019 epidemic
**3**	"This is a mixed method study combining quantitative and qualitative methods, It's unclear why the Authors did not mention this."	"The study utilized a mixed-methods approach..."	"This is a mixed-methods study combining quantitative household surveys with qualitative key informant interviews to provide a comprehensive evaluation."	Specified the mixed-methods study design at the outset to ensure immediate methodological clarity and transparency for the reader
**4**	"The Author may take a look into the sampling technique... or it's a multistage sampling technique."	"The sampling method used was a two-stage stratified cluster random sampling."	"A two-stage stratified cluster random sampling technique was employed, which constitutes a multistage sampling approach."	Clarified the two-stage sampling procedure to explicitly confirm that the design employs a standard multistage cluster random sampling approach
**5**	"Measures taken to minimize bias... particularly the qualitative data."	"Qualitative interviews were conducted with health system stakeholders..."	"To ensure the scientific rigor and credibility of qualitative findings and minimize bias, triangulation was employed by interviewing diverse stakeholder groups... data were analyzed using thematic content analysis following a formal four-step framework..."	Incorporated specific details on data triangulation and the systematic analysis framework to enhance the methodological robustness, validity, and replicability of the qualitative component

Table 2: Addressing Comments from Reviewer 2 (Major Comments)

N°	Examiner comment (Full Quote)	Original sentence/phrase (in the manuscript)	New/improved sentence/phrase	Justification / rationale
**1**	"Clearly explain how vaccination status was determined (vaccination cards, caregiver report, etc.)."	"Possession of a vaccination card was	"Vaccination status was determined based on a combination of documented evidence from	Specified the data source to allow for a more robust assessment of

		neither an inclusion nor exclusion criterion."	vaccination cards (seen by surveyors) and caregiver recall for children without cards."	potential recall or misclassification bias
**2**	"Clarify whether the multivariate logistic regression models accounted for the complex survey design (clustering, stratification, and weighting)."	"Logistic models were used to identify the determinants..."	"Multivariate logistic regression models were constructed using survey-set (svy) commands in STATA to account for the complex survey design, including clustering, stratification, and sampling weights."	Clarified the statistical approach to ensure correct estimation of standard errors and confidence intervals for the complex survey design
**3**	"Clearly define the criteria used to classify districts into each stratum (large city, landlocked, intermediate)."	"Three strata were defined a priori to reduce variance and increase the precision..."	"Districts were classified into: (1) large city districts based on urban density; (2) landlocked districts characterized by limited road access and significant geographic barriers; and (3) intermediate districts."	Defined the classification criteria to better support the interpretation of geographic disparities
**4**	"The methods state that insecurity zones were excluded... discuss how they may affect the representativeness."	"excluded insecurity zones before the selection of the enumeration zones"	"The exclusion of three enumeration areas due to insecurity represents a limitation, although post-stratified adjustments were applied to correct the weights."	Clarified the extent of geographical exclusions to provide a more transparent assessment of the national estimates' scope
**5**	"Report the number and type of participants interviewed... how they were selected, and how data were analysed."	"Qualitative interviews were conducted with health system stakeholders..."	"Qualitative data were gathered through semi-structured interviews with 227 health system stakeholders (61 regional and district health officials and 166 community leaders and agents) selected through purposive sampling..."	Added specific stakeholder counts and selection methods to strengthen the rigor of mixed-methods reporting
**6**	"Adjusted odds ratios should be presented with 95% confidence intervals, not only p-values."	(Table 2 originally only reported AOR and p-values)	Updated Table 2 to include "95% Confidence Interval (95% CI)" columns for all variables.	Included 95% Confidence Intervals for all variables to allow for assessment of precision and significance beyond p-values

Table 3: Addressing Comments from Academic Editor and Minor Comments

N°	Examiner comment (Full Quote)	Original sentence/phrase (in the manuscript)	New/improved sentence/phrase	Justification / rationale
1	"Consolidating the discussion of study limitations into a dedicated limitations section within the Discussion."	(Found in Materials and Methods section in the original version)	"Study strengths and limitations" (Section relocated and consolidated within the Discussion)	Relocated study limitations to the Discussion to focus the methodology on procedures and follow standard academic conventions

2	"Specify the qualitative methodology and analytical approach used... Providing sufficient methodological detail is essential."	"Qualitative interviews were conducted with health system stakeholders..."	"Qualitative data were analysed using thematic content analysis following a formal four-step framework: (1) transcription and familiarization; (2) formal coding; (3) development of an analytical framework; and (4) thematic interpretation."	Added a formal analysis framework to provide the transparency required to assess the rigor and validity of the qualitative component
3	"The abstract uses the term 'vaccination intention,' while the methods focus on actual post-campaign vaccination coverage. Use consistent terminology."	"...were associated with increased vaccination intention."	"...were associated with significantly higher post-campaign vaccination coverage."	Replacing "intention" with "coverage" accurately reflects that the study measured actual vaccine receipt during the campaign.
4	"The final number of eligible children included in the analysis should be clearly reported, overall and by stratum."	"revealed a national vaccination coverage of 75.3%."	"The study included 1,294 eligible children (Large cities: n=363; Landlocked: n=410; Intermediate: n=521). The national post-campaign vaccination coverage was 75.3%."	Reported denominators (n) for all geographic strata to enhance statistical transparency and clarity
5	"It would be useful to specify which software was used for the main statistical analyses."	"analysed using Excel, SPSS and STATA software."	"STATA (version 17.0) was used for all main statistical analyses, including multivariate modeling and adjustments for the complex survey design."	Specified STATA (version 17.0) for the main analysis to address the editorial request regarding advanced modeling software
6	"The methods section could be slightly streamlined to avoid repetition regarding the study period..."	(Dates repeated in paragraphs 8 and 9)	"The vaccination campaign period was October 2024." (Redundant study duration dates removed from paragraph 9).	Streamlined the Methods section by removing redundant dates to improve the overall flow of the manuscript
7	"Please ensure that your Ethics Statement is available in its entirety at the beginning of your Methods section, under a subheading 'Ethics Statement'."	(The statement was originally located at the end of the Materials and Methods section)	"Materials and methods / Ethics Statement / Ethical approval was granted by... (Ref: 021/2024-CEERINSPC/IRB)."	Relocated the Ethics Statement to the beginning of the Methods section to comply with the Editor's specific structural requirements
8	"Please upload separate figure files in .tif or .eps format. Also, remove the figures from your manuscript file but keep the legends."	"Figure 1: Post-campaign measles vaccination coverage by stratum (2024)"	"Fig 1: Post-campaign measles vaccination coverage by stratum (2024)" (Images removed; text captions retained using the "Fig" abbreviation)	Followed journal policy by removing figure images from the manuscript while providing high-resolution files separately
9	"Label: Use Arabic numerals and abbreviate 'Figure' to 'Fig'... Title:	"Figure 2: Determinants of post-campaign vaccination intention"	"Fig 2: Determinants of post-campaign vaccination coverage"	Updated figure labels and titles for conciseness (under the 15-word

	This should be a concise description of 15 words or less."			limit) and terminological consistency throughout the manuscript.
