# Supplementary material for: Determinants of post-campaign measles vaccination coverage in Madagascar: A geographically stratified analysis
Source: PLOS Glob Public Health. 2026 Aug 21;6(8):e0006390. doi: 10.1371/journal.pgph.0006390 (PMC13496406; doi:10.1371/journal.pgph.0006390)
Supplement: Supplementary file 2 [file pgph.0006390.s004.pdf]

Dr. Félix ALAIN,
Corresponding Author,
Antananarivo, Madagascar
E-mail: alainfelixmed@gmail.com

Antananarivo, July 15th, 2026

To: Editorial Office, PLOS Global Public Health

**<u>Subject:</u> Resubmission of Revised Manuscript PGPH-D-26-01253R2**

**Title:** Determinants of post-campaign measles vaccination coverage in Madagascar: A geographically stratified analysis

Dear Editor,

We would like to thank the editors and the reviewers for their constructive feedback on our manuscript. We have carefully addressed all the requested revisions to improve the clarity, context, and methodological rigour of our study.
As requested, we are submitting the following documents:

1. This point-by-point response document;
2. A marked-up copy of the manuscript with track changes;
3. A "Clean Copy" of the revised manuscript.

We believe these changes have significantly strengthened the paper and look forward to your decision.
Sincerely,

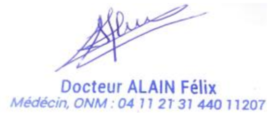


Table N°1: Addressing Comments from the Academic Editor

| Examiner comment (Full Quote) | Original sentence/phrase (in the manuscript) | New/improved sentence/phrase | Justification / rationale |
|---|---|---|---|
| "There is still uncertainty around the qualitative approach in this study. You've stated that you followed a | "qualitative data were analysed using thematic content analysis following | "Qualitative data underwent thematic content analysis **adhering to the Framework Method (e.g. Ritchie & Spencer)**, comprising: (1) | Providing a formal academic reference for the qualitative analysis methodology |

| | | | |
|---|---|---|---|
| framework, but there is no reference to which one. If no published framework was followed, this must be included in the limitations section." | a formal four-step framework: (1) transcription and familiarization... (2) formal coding... (3) development and application of an analytical framework... and (4) thematic interpretation..." | transcription and **familiarisation**; (2) **systematic** coding; (3) **analytical** framework development; and (4) thematic **synthesis** to identify constants and relationships between participants." | ensures **methodological rigour** and replicability as requested by the Editor. |
| "Please check the previous report... including the inclusion of reporting guidelines for qualitative research. In particular, consider using the Consolidated Criteria for Reporting Qualitative Research (COREQ) and providing the completed checklist as a supplementary file." | (This statement was previously absent from the methodology section). | "The qualitative investigation and reporting followed the Consolidated Criteria for Reporting Qualitative Research (COREQ). The complete 32-item checklist is included as **S1 File**." | Adherence to COREQ standards ensures transparency in reporting qualitative research and complies with the journal's methodological requirements. |
| "It is unclear whether a formal mixed methods methodology was employed... I recommend explicitly describing the mixed methods design, rationale, and integration procedures." | "This is a mixed-methods study combining quantitative household surveys with qualitative interviews to analyse the performance of the measles vaccination campaign..." | "A **convergent parallel mixed-methods design** was utilised, wherein quantitative household surveys and **semistructured** qualitative interviews were conducted concurrently. Data were integrated during the interpretation phase to **triangulate** statistical determinants with contextual stakeholder insights." | The revision clarifies the specific mixed-methods typology used and provides a scientific rationale for the integration of disparate data types. |
| "I would also hope to see the integration of the quantitative and qualitative findings in the discussion, at a minimum." | "Qualitative data highlighted religious/socio-cultural beliefs, fear of side effects, and insufficient | "The identified statistical determinants, such as the lower coverage among urban residents (**ORa = 0.25**), were **contextualised** using qualitative data, which highlighted that 'urban vaccine hesitancy' is exacerbated by | **Synthesis** of findings within the discussion demonstrates methodological integration and provides a more robust |

| | | | |
|---|---|---|---|
| | information as key reasons for non-vaccination." | metropolitan misinformation and perceptions that previously vaccinated children did not require campaign doses." | interpretation of the determinants. |

Table N°2: Addressing Comments from Reviewer #1 (Sunday Charles Adeyemo)

| **Examiner comment (Full Quote)** | **Original sentence/phrase (in the manuscript)** | **New/improved sentence/phrase** | **Justification / rationale** |
|---|---|---|---|
| "I am still of the view that the results of the qualitative components can be made more transparent and be better integrated into the discussion." | "Qualitative interviews with community leaders and health facility staff provided deeper insights into campaign implementation and community perceptions." | "To enhance transparency, qualitative findings from **227 stakeholders** were **synthesised** within the Discussion to **contextualise** quantitative determinants; for instance, the success of community-led recruitment in addressing identified 'hostility towards vaccinators' provides a strategy to **mitigate** the barriers observed in large cities." | The revision addresses the reviewer's concern regarding transparency by more explicitly linking qualitative evidence to the statistical determinants discussed. |

Table N°3: Addressing Comments from Reviewer #2

| **Examiner comment (Full Quote)** | **Original sentence/phrase (in the manuscript)** | **New/improved sentence/phrase** | **Justification / rationale** |
|---|---|---|---|
| "The authors have adequately addressed my previous comments and revised the manuscript accordingly. I have no further concerns and support the manuscript's acceptance." | - | - | The reviewer expressed full satisfaction with the current state of the manuscript and recommended its acceptance for publication. |
