# Supplementary material for: Determinants of post-campaign measles vaccination coverage in Madagascar: A geographically stratified analysis
Source: PLOS Glob Public Health. 2026 Aug 21;6(8):e0006390. doi: 10.1371/journal.pgph.0006390 (PMC13496406; doi:10.1371/journal.pgph.0006390)
Supplement: Supplementary file 3 [file pgph.0006390.s005.pdf]

Dr. Félix ALAIN,
Corresponding Author,
Antananarivo, Madagascar
E-mail: alainfelixmed@gmail.com

Antananarivo, July 20th, 2026

To: Editorial Office, PLOS Global Public Health

**Subject: Resubmission of Revised Manuscript [PGPH-D-26-01253R2]**

**Title:** "Determinants of post-campaign measles vaccination coverage in Madagascar: A geographically stratified analysis"

Dear Editor,

We would like to thank the Academic Editor, Dr. Matthew N. Berger, for the constructive final feedback on our manuscript. We have carefully addressed the remaining minor points to ensure methodological transparency and to meet the journal's high publication standards.

As requested, we are submitting the following documents as part of this revision:

1. This Response to Reviewers (Point-by-point response);
2. A Revised Manuscript with Track Changes highlighting all modifications;
3. A clean, unmarked version of the Manuscript;
4. An updated COREQ Checklist (S1 File).

We believe these refinements have further strengthened the clarity and rigour of our report.

Sincerely,

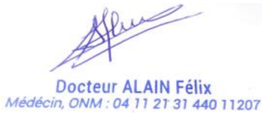


**Table 1: Response to Academic Editor Comments**

| Examiner comment (Full Quote) | Original sentence/phrase (in the manuscript) | New/improved sentence/phrase | Justification / rationale |
|---|---|---|---|
| "Could you please review the references and ensure that the COREQ guideline is cited in the manuscript: Tong A, Sainsbury P, Craig J. Consolidated criteria for reporting qualitative research (COREQ): a 32-item | "The qualitative investigation and reporting followed the Consolidated Criteria for Reporting Qualitative | "The qualitative investigation and reporting followed the Consolidated Criteria for Reporting Qualitative Research (COREQ) **[14].** The | The specific reference for the COREQ guidelines (Tong et al., 2007) has been added to the manuscript and the reference list to ensure methodological transparency as requested. |

| | | | |
|---|---|---|---|
| checklist for interviews and focus groups. International Journal for Quality in Health Care. 2007;19(6):349–357. doi:10.1093/intqhc/mzm042." | Research (COREQ). The complete 32-item checklist is included as S1 File." | complete 32-item checklist is included as S1 File." | |
| "The manuscript also refers to “(e.g., Ritchie & Spencer).” Please clarify whether the research team formally followed the Ritchie and Spencer framework during the analysis... If the framework was formally used, please remove “e.g.,” and include the appropriate reference within the manuscript." | "...qualitative data underwent thematic content analysis adhering to the Framework Method (e.g. Ritchie & Spencer), comprising: (1) transcription and familiarisation..." | "...qualitative data underwent thematic content analysis adhering to the Framework Method **(Ritchie & Spencer) [13]**, comprising: (1) transcription and familiarisation..." | We have confirmed that the Ritchie and Spencer framework was formally utilised for the thematic analysis. Consequently, "e.g." has been removed and the formal reference has been included as requested by the Editor. |
| "Please review your reference list to ensure that it is complete and correct. If you have cited papers that have been retracted, please include the rationale for doing so... or remove these references..." | (General Reference List) | (Updated Reference List) | A thorough review of the bibliography was conducted. All references were checked for accuracy, and we have verified that no retracted articles are included in the final list. |
| "While revising your submission, we strongly recommend that you use PLOS’s NAAS tool... to test your figure files." | N/A | (Uploaded NAAS-processed figure files: Figure1.tif and Figure2.tif) | All figures **have been processed** through the PLOS NAAS tool. The tool successfully adjusted the resolution from 96 DPI to 300 DPI and corrected the dimensions to meet the journal's technical requirements. Both files are now marked as "fixed". |
