# Supplementary material for: Determinants of post-campaign measles vaccination coverage in Madagascar: A geographically stratified analysis
Source: PLOS Glob Public Health. 2026 Aug 21;6(8):e0006390. doi: 10.1371/journal.pgph.0006390 (PMC13496406; doi:10.1371/journal.pgph.0006390)
Supplement: Supplementary file 4 [file pgph.0006390.s001.docx]

**S1 File**

**COREQ: 32-Item Checklist for Qualitative Research**

*************************

Title of study: "**Determinants of post-campaign measles vaccination coverage in Madagascar: A geographically stratified analysis**"

| **Item No.** | **Response** |  |
| --- | --- | --- |
| **Domain 1: Research team and reflexivity** | | |
| **Personal Characteristics** | | |
| 1. Interviewer/facilitator | Interviews were conducted by supervisors and researchers from the National Institute of Public and Community Health (INSPC) |  |
| 2. Credentials | The research was carried out by a multi-disciplinary team, including medical doctors (MD), MPH, Epidemiologist, Anthropologist, Biostatisticians and PhD in Public Health |  |
| 3. Occupation | The team included the Director General of the INSPC, technical coordinators, point focals, data managers, and researchers |  |
| 4. Gender | The multi-disciplinary team comprised both male and female researchers. |  |
| 5. Experience and training | Researchers were experienced in the field and trained specifically in qualitative and quantitative contexts and the use of collection tools. |  |
| **Relationship with participants** | | |
| 6. Relationship established | Participants were selected through purposive sampling; no prior relationship was explicitly reported before the study. |  |
| 7. Participant knowledge of the interviewer | Participants were informed of the study’s goals and reasons for research during the informed consent process |  |
| 8. Interviewer characteristics | The researchers were reported to have expertise in qualitative contexts to ensure methodological rigor and minimize bias |  |
| **Domain 2: Study design** | | |
| **Theoretical framework** | | |
| 9. Methodological orientation and Theory | The study employed thematic content analysis adhering to the Framework Method within a mixed-methods study design |  |
| **Participant selection** | | |
| 10. Sampling | Purposive sampling was employed to ensure diverse representation across different levels of the health system. |  |
| 11. Method of approach | Stakeholders were approached directly for semi-structured interviews. |  |
| 12. Sample size | The study included 227 health system stakeholders, including 61 regional and district officials and 166 community leaders and agents. |  |
| 13. Non-participation | The household survey had a 99.3% acceptance rate; qualitative participation was comprehensive with no reported dropouts. |  |
| **Setting** | | |
| 14. Setting of data collection | Data was collected across all 23 regions of Madagascar at various health system levels. |  |
| 15. Presence of non-participants | No non-participants were reported to be present during the data collection. |  |
| 16. Description of sample | The sample included regional and district health officials, community leaders, and community agents. |  |
| **Data collection** | | |
| 17. Interview guide | Semi-structured interview guides were developed by the authors and pilot-tested before use. |  |
| 18. Repeat interviews | No repeat interviews were carried out. |  |
| 19. Audio/visual recording | Audio recordings were used to collect qualitative data during the interviews to ensure accuracy |  |
| 20. Field notes | Interviewer journals and field reports were utilised to record observations and track progress. |  |
| 21. Duration | Standard durations for semi-structured interviews were employed. |  |
| 22. Data saturation | Data triangulation across diverse groups (officials vs. community agents) was utilized to ensure the validity and saturation of findings. |  |
| 23. Transcripts returned | Transcripts were not returned to participants for comment. |  |
| **Domain 3: Analysis and findings** | | |
| **Data analysis** | | |
| 24. Number of data coders | Dedicated analysis workshops involving the multi-disciplinary research team were conducted for cross-sectional review and transcription completion. |  |
| 25. Description of the coding tree | Formal coding was used to organize data units systematically into categories. |  |
| 26. Derivation of themes | Themes were derived using the Framework Method (Ritchie & Spencer) comprising: (1) transcription/familiarization; (2) formal coding; (3) development of an analytical framework; and (4) thematic interpretation. |  |
| 27. Software | Excel, SPSS, and STATA (version 17.0) were used for data cleaning and analysis, with qualitative thematic synthesis managed in Excel. |  |
| 28. Participant checking | No participant feedback on the final findings was reported. |  |
| **Reporting** |  |  |
| 29. Quotations presented | Relevant findings are illustrated through verbatims (direct quotations) in the results. |  |
| 30. Data and findings consistent | Consistency was ensured through triangulation of data across diverse stakeholder groups. |  |
| 31. Clarity of major themes | Major themes, including reasons for refusal, campaign coordination, and communication gaps, are clearly presented. |  |
| 32. Clarity of minor themes | Minor themes such as waste management and specific logistical supply chain challenges are discussed. |  |
