# Supplementary material for: Determinants of post-campaign measles vaccination coverage in Madagascar: A geographically stratified analysis
Source: PLOS Glob Public Health. 2026 Aug 21;6(8):e0006390. doi: 10.1371/journal.pgph.0006390 (PMC13496406; doi:10.1371/journal.pgph.0006390)
Supplement: Peer review history

# Peer review: Decision Letter 0

**DOI:** 10.1371/journal.pgph.0006390.r001

**Authors:** Matthew Berger

31 May 2026

PGPH-D-26-01253

Analysis of the determinants of post-campaign measles vaccination coverage in Madagascar: A stratified approach by geographical areas

PLOS Global Public Health

Dear Dr. ALAIN,

Thank you for submitting your manuscript to PLOS Global Public Health. After careful consideration, we feel that it has merit but does not fully meet PLOS Global Public Health’s publication criteria as it currently stands. Therefore, we invite you to submit a revised version of the manuscript that addresses the points raised during the review process.

Please submit your revised manuscript by Jul 30 2026 11:59PM. If you will need more time than this to complete your revisions, please reply to this message or contact the journal office at globalpubhealth@plos.org. When you're ready to submit your revision, log on to https://www.editorialmanager.com/pgph/ and select the 'Submissions Needing Revision' folder to locate your manuscript file.

Please include the following items when submitting your revised manuscript:

- A letter that responds to each point raised by the editor and reviewer(s). You should upload this letter as a separate file labeled 'Response to Reviewers'.
- A marked-up copy of your manuscript that highlights changes made to the original version. You should upload this as a separate file labeled 'Revised Manuscript with Track Changes'.
- An unmarked version of your revised paper without tracked changes. You should upload this as a separate file labeled 'Manuscript'.

Guidelines for resubmitting your figure files are available below the reviewer comments at the end of this letter.

As the corresponding author, your ORCID iD is verified in the submission system and will appear in the published article. PLOS supports the use of ORCID, and we encourage all coauthors to register for an ORCID iD and use it as well. Please encourage your coauthors to verify their ORCID iD within the submission system before final acceptance, as unverified ORCID iDs will not appear in the published article. *Only*  the individual author can complete the verification step; PLOS staff *cannot*  verify ORCID iDs on behalf of authors.

We look forward to receiving your revised manuscript.

Kind regards,

Matthew N. Berger, PhD

Academic Editor

PLOS Global Public Health

**Journal Requirements:**

1. Please amend your detailed Financial Disclosure statement. This is published with the article. It must therefore be completed in full sentences and contain the exact wording you wish to be published.

i. Please clarify all sources of financial support for your study. List the grants, grant numbers, and organizations that funded your study, including funding received from your institution. Please note that suppliers of material support, including research materials, should be recognized in the Acknowledgements section rather than in the Financial Disclosure.

ii. State the initials, alongside each funding source, of each author to receive each grant. For example: "This work was supported by the National Institutes of Health (####### to AM; ###### to CJ) and the National Science Foundation (###### to AM)."

iii. State what role the funders took in the study. If the funders had no role in your study, please state: “The funders had no role in study design, data collection and analysis, decision to publish, or preparation of the manuscript.”

iv. If any authors received a salary from any of your funders, please state which authors and which funders.

2. We note that your Data Availability Statement is currently as follows: All relevant data underlying the findings of this study are available within the manuscript and its Supporting Information files.

Please confirm at this time whether or not your submission contains all raw data required to replicate the results of your study. Authors must share the “minimal data set” for their submission. PLOS defines the minimal data set to consist of the data required to replicate all study findings reported in the article, as well as related metadata and methods (https://journals.plos.org/plosone/s/data-availability#loc-minimal-data-set-definition).

For example, authors should submit the following data:

- The values behind the means, standard deviations and other measures reported;

- The values used to build graphs;

- The points extracted from images for analysis.

Authors do not need to submit their entire data set if only a portion of the data was used in the reported study.

If your submission does not contain these data, please either upload them as Supporting Information files or deposit them to a stable, public repository and provide us with the relevant URLs, DOIs, or accession numbers. For a list of recommended repositories, please see https://journals.plos.org/plosone/s/recommended-repositories.

If there are ethical or legal restrictions on sharing a de-identified data set, please explain them in detail (e.g., data contain potentially sensitive information, data are owned by a third-party organization, etc.) and who has imposed them (e.g., an ethics committee). Please also provide contact information for a data access committee, ethics committee, or other institutional body to which data requests may be sent. If data are owned by a third party, please indicate how others may request data access.

3. We note that you have indicated that there are restrictions to data sharing for this study. For studies involving human research participant data or other sensitive data, we encourage authors to share de-identified or anonymized data. However, when data cannot be publicly shared for ethical reasons, we allow authors to make their data sets available upon request. For information on unacceptable data access restrictions, please see http://journals.plos.org/plosone/s/data-availability#loc-unacceptable-data-access-restrictions.

Before we proceed with your manuscript, please address the following prompts:

a) If there are ethical or legal restrictions on sharing a de-identified data set, please explain them in detail (e.g., data contain potentially identifying or sensitive patient information, data are owned by a third-party organization, etc.) and who has imposed them (e.g., a Research Ethics Committee or Institutional Review Board, etc.). Please also provide contact information for a data access committee, ethics committee, or other institutional body to which data requests may be sent.

b) If there are no restrictions, please upload the minimal anonymized data set necessary to replicate your study findings to a stable, public repository and provide us with the relevant URLs, DOIs, or accession numbers. Please see http://www.bmj.com/content/340/bmj.c181.long for guidelines on how to de-identify and prepare clinical data for publication. For a list of recommended repositories, please see https://journals.plos.org/plosone/s/recommended-repositories. You also have the option of uploading the data as Supporting Information files, but we would recommend depositing data directly to a data repository if possible.

Please update your Data Availability statement in the submission form accordingly.

4. Please upload separate figure files in .tif or .eps format. Also, remove the figures from your manuscript file but keep the legends.

For more information about figure files please see our guidelines:

https://journals.plos.org/globalpublichealth/s/figures

https://journals.plos.org/globalpublichealth/s/figures#loc-file-requirements

5. Please ensure that your Ethics Statement is available in its entirety at the beginning of your Methods section, under a subheading 'Ethics Statement'.

If the reviewer comments include a recommendation to cite specific previously published works, please review and evaluate these publications to determine whether they are relevant and should be cited. There is no requirement to cite these works unless the editor has indicated otherwise.

**Additional Editor Comments (if provided):**

Academic Editor

1. I recommend consolidating the discussion of study limitations into a dedicated limitations section within the Discussion. The current limitations presented in the Methods section could be moved and streamlined to avoid repetition and improve the overall flow of the manuscript.

2. The qualitative component of the study requires further clarification. Please specify the qualitative methodology and analytical approach used (e.g., thematic analysis, content analysis, grounded theory, framework analysis, or another method). Providing sufficient methodological detail is essential for readers to assess the rigour and trustworthiness of the findings.

3. I strongly recommend reviewing established reporting guidelines for qualitative research. In particular, consider using the Consolidated Criteria for Reporting Qualitative Research (COREQ) and providing the completed checklist as a supplementary file. Alternatively, the Standards for Reporting Qualitative Research (SRQR) may be appropriate. Adherence to one of these reporting frameworks would substantially improve transparency and reporting quality.

4. It is unclear whether a formal mixed methods methodology was employed. As currently presented, the manuscript appears more consistent with a multi-method study, where quantitative and qualitative components are conducted alongside one another but without clear methodological integration. If the intention is to present this as a mixed methods study, I recommend explicitly describing the mixed methods design, rationale, and integration procedures. The following resource may be helpful: Poth, C. N. (Ed.). (2024). The Sage Handbook of Mixed Methods Research Design.

5. If this manuscript is intended to be reported as a mixed methods study, greater integration of the quantitative and qualitative findings is needed. At a minimum, the Discussion should explicitly bring together findings from both components, highlighting areas of convergence, complementarity, or divergence. Strengthening this integration would enhance the overall interpretation of the results and demonstrate the added value of employing both quantitative and qualitative approaches.

Reviewer 1

1) As a reviewer I am of the view that your topic is clear and research-worthy but the phrasing can be tightened for structure and clarity I would suggest the following

- Determinants of post-campaign measles vaccination coverage in Madagascar; A geographically stratified analysis.

-Geographic variation in determinants of measles vaccination coverage following supplementary supplementary immunization campaign in Madagascar.

- What drives post-campaign measles vaccination coverage in Madagascar, A subnational stratified analysis.

2)It would not be out of place to discuss the geography of Madagascar, sociodemographic features, what has been done previously in immunization and post immunization campaigns as these would mirror the study much better and improve the justification for study.

3)This is a mixed method study combining quantitative and qualitative methods, It’s unclear why the Authors did not mention this in the manuscript.

4)The Author may take a look into the sampling technique if it’s exactly what it was said to be or it’s a multistage sampling technique.

5)It would be good if measures taken to minimize bias and chance are included in the manuscript particularly the qualitative data so as to improve the validity of the study and replicability of the methodology.

Reviewer 2

The manuscript addresses an important and timely public health issue: post-campaign measles vaccination coverage among children aged 9–59 months in Madagascar following the October 2024 vaccination campaign. The topic is clearly relevant to global public health, and the study has several strengths, including national coverage across all 23 regions, a stratified cluster sampling design, and the use of both quantitative and qualitative data.

However, several methodological and reporting issues require clarification before the findings can be fully assessed.

Major comments

1. Assessment of vaccination status

The manuscript states that post-campaign vaccination coverage was assessed, but the methods should more clearly explain how vaccination status was determined for each eligible child. It is not clear whether this was based on vaccination cards, campaign documentation, finger marking, caregiver report, or a combination of sources. This is important for assessing the reliability of the coverage estimates and the potential for recall or misclassification bias.

2. Statistical analysis and survey design

The sampling strategy and weighting procedure are described, which is useful. However, the authors should clarify whether the multivariate logistic regression models accounted for the complex survey design, including clustering, stratification, and sampling weights. This is important for the correct estimation of standard errors, confidence intervals, and statistical significance.

3. Definition of geographic strata

The manuscript classifies districts into three strata: large city districts, landlocked districts, and intermediate districts. Since these categories are central to the analysis and interpretation of geographic disparities, the authors should clearly define the criteria used to classify districts into each stratum and explain the rationale for this classification.

4. Exclusion of insecurity zones

The methods state that insecurity zones were excluded from the sampling frame before the selection of enumeration areas. The authors should clarify the extent of these exclusions and discuss how they may affect the representativeness of the national coverage estimates. This is particularly important if excluded areas may have different access to vaccination services or lower campaign coverage.

5. Qualitative component

The qualitative component is relevant for understanding reasons for non-vaccination, but the methods provide limited detail. The authors should report the number and type of participants interviewed, how they were selected, and how the qualitative data were analysed. A clearer description would strengthen the mixed-methods aspect of the study.

6. Comparison with 2022 coverage

The abstract reports an increase in coverage from 65.3% in 2022 to 75.3% in 2024. The authors should clarify whether the 2022 and 2024 estimates are directly comparable in terms of survey methods, target population, sampling frame, and definition of vaccination coverage. If there were methodological differences, the comparison should be interpreted more cautiously.

Minor comments

1. The abstract uses the term “vaccination intention,” while the methods appear to focus on actual post-campaign vaccination coverage. The authors should use consistent terminology throughout the manuscript.

2. The final number of eligible children included in the analysis should be clearly reported, overall and by stratum.

3. Adjusted odds ratios should be presented with 95% confidence intervals, not only p-values.

4. The authors mention using Excel, SPSS, and STATA. It would be useful to specify which software was used for the main statistical analyses.

5. The methods section could be slightly streamlined to avoid repetition regarding the study period and data collection period.

Overall recommendation

The manuscript addresses a relevant and important topic and has the potential to provide useful evidence for immunization planning in Madagascar. The national scope of the survey and the mixed-methods approach are important strengths. However, several methodological and reporting issues require clarification, particularly regarding the assessment of vaccination status, the treatment of the survey design in the analysis, the exclusion of insecurity zones, and the qualitative methods.

[Note: HTML markup is below. Please do not edit.]

Reviewers' comments:

Reviewer's Responses to Questions

**Comments to the Author**

1. Does this manuscript meet PLOS Global Public Health’s publication criteria? Is the manuscript technically sound, and do the data support the conclusions? The manuscript must describe methodologically and ethically rigorous research with conclusions that are appropriately drawn based on the data presented.

Reviewer #1: Partly

Reviewer #2: Partly

**********

2. Has the statistical analysis been performed appropriately and rigorously?

Reviewer #1: Yes

Reviewer #2: Yes

**********

3. Have the authors made all data underlying the findings in their manuscript fully available (please refer to the Data Availability Statement at the start of the manuscript PDF file)?

The PLOS Data policy requires authors to make all data underlying the findings described in their manuscript fully available without restriction, with rare exception. The data should be provided as part of the manuscript or its supporting information, or deposited to a public repository. For example, in addition to summary statistics, the data points behind means, medians and variance measures should be available. If there are restrictions on publicly sharing data—e.g. participant privacy or use of data from a third party—those must be specified.

Reviewer #1: Yes

Reviewer #2: Yes

**********

4. Is the manuscript presented in an intelligible fashion and written in standard English?

PLOS Global Public Health does not copyedit accepted manuscripts, so the language in submitted articles must be clear, correct, and unambiguous. Any typographical or grammatical errors should be corrected at revision, so please note any specific errors here.

Reviewer #1: Yes

Reviewer #2: Yes

**********

5. Review Comments to the Author

Please use the space provided to explain your answers to the questions above. You may also include additional comments for the author, including concerns about dual publication, research ethics, or publication ethics. (Please upload your review as an attachment if it exceeds 20,000 characters)

**Reviewer #1:**  1) As a reviewer I am of the view that your topic is clear and research-worthy but the phrasing can be tightened for structure and clarity I would suggest the following

- Determinants of post-campaign measles vaccination coverage in Madagascar; A geographically stratified analysis.

-Geographic variation in determinants of measles vaccination coverage following supplementary supplementary immunization campaign in Madagascar.

- What drives post-campaign measles vaccination coverage in Madagascar, A subnational stratified analysis.

2)It would not be out of place to discuss the geography of Madagascar, sociodemographic features, what has been done previously in immunization and post immunization campaigns as these would mirror the study much better and improve the justification for study.

3)This is a mixed method study combining quantitative and qualitative methods, It’s unclear why the Authors did not mention this in the manuscript.

4)The Author may take a look into the sampling technique if it’s exactly what it was said to be or it’s a multistage sampling technique.

5)It would be good if measures taken to minimize bias and chance are included in the manuscript particularly the qualitative data so as to improve the validity of the study and replicability of the methodology.

**Reviewer #2:**  Reviewer comments

The manuscript addresses an important and timely public health issue: post-campaign measles vaccination coverage among children aged 9–59 months in Madagascar following the October 2024 vaccination campaign. The topic is clearly relevant to global public health, and the study has several strengths, including national coverage across all 23 regions, a stratified cluster sampling design, and the use of both quantitative and qualitative data.

However, several methodological and reporting issues require clarification before the findings can be fully assessed.

Major comments

1. Assessment of vaccination status

The manuscript states that post-campaign vaccination coverage was assessed, but the methods should more clearly explain how vaccination status was determined for each eligible child. It is not clear whether this was based on vaccination cards, campaign documentation, finger marking, caregiver report, or a combination of sources. This is important for assessing the reliability of the coverage estimates and the potential for recall or misclassification bias.

2. Statistical analysis and survey design

The sampling strategy and weighting procedure are described, which is useful. However, the authors should clarify whether the multivariate logistic regression models accounted for the complex survey design, including clustering, stratification, and sampling weights. This is important for the correct estimation of standard errors, confidence intervals, and statistical significance.

3. Definition of geographic strata

The manuscript classifies districts into three strata: large city districts, landlocked districts, and intermediate districts. Since these categories are central to the analysis and interpretation of geographic disparities, the authors should clearly define the criteria used to classify districts into each stratum and explain the rationale for this classification.

4. Exclusion of insecurity zones

The methods state that insecurity zones were excluded from the sampling frame before the selection of enumeration areas. The authors should clarify the extent of these exclusions and discuss how they may affect the representativeness of the national coverage estimates. This is particularly important if excluded areas may have different access to vaccination services or lower campaign coverage.

5. Qualitative component

The qualitative component is relevant for understanding reasons for non-vaccination, but the methods provide limited detail. The authors should report the number and type of participants interviewed, how they were selected, and how the qualitative data were analysed. A clearer description would strengthen the mixed-methods aspect of the study.

6. Comparison with 2022 coverage

The abstract reports an increase in coverage from 65.3% in 2022 to 75.3% in 2024. The authors should clarify whether the 2022 and 2024 estimates are directly comparable in terms of survey methods, target population, sampling frame, and definition of vaccination coverage. If there were methodological differences, the comparison should be interpreted more cautiously.

Minor comments

1. The abstract uses the term “vaccination intention,” while the methods appear to focus on actual post-campaign vaccination coverage. The authors should use consistent terminology throughout the manuscript.

2. The final number of eligible children included in the analysis should be clearly reported, overall and by stratum.

3. Adjusted odds ratios should be presented with 95% confidence intervals, not only p-values.

4. The authors mention using Excel, SPSS, and STATA. It would be useful to specify which software was used for the main statistical analyses.

5. The methods section could be slightly streamlined to avoid repetition regarding the study period and data collection period.

Overall recommendation

The manuscript addresses a relevant and important topic and has the potential to provide useful evidence for immunization planning in Madagascar. The national scope of the survey and the mixed-methods approach are important strengths. However, several methodological and reporting issues require clarification, particularly regarding the assessment of vaccination status, the treatment of the survey design in the analysis, the exclusion of insecurity zones, and the qualitative methods.

**********

6. PLOS authors have the option to publish the peer review history of their article (what does this mean?). If published, this will include your full peer review and any attached files.

**Do you want your identity to be public for this peer review?**  If you choose “no”, your identity will remain anonymous but your review may still be made public.

For information about this choice, including consent withdrawal, please see our Privacy Policy.

Reviewer #1: **Yes:**  Sunday charles Adeyemo

Reviewer #2: No

**********

[NOTE: If reviewer comments were submitted as an attachment file, they will be attached to this email and accessible via the submission site. Please log into your account, locate the manuscript record, and check for the action link "View Attachments". If this link does not appear, there are no attachment files.]

**Figure Resubmissions:**

While revising your submission, we strongly recommend that you use PLOS’s NAAS tool (https://ngplosjournals.pagemajik.ai/artanalysis) to test your figure files. NAAS can convert your figure files to the TIFF file type and meet basic requirements (such as print size, resolution), or provide you with a report on issues that do not meet our requirements and that NAAS cannot fix.

After uploading your figures to PLOS’s NAAS tool - https://ngplosjournals.pagemajik.ai/artanalysis, NAAS will process the files provided and display the results in the "Uploaded Files" section of the page as the processing is complete. If the uploaded figures meet our requirements (or NAAS is able to fix the files to meet our requirements), the figure will be marked as "fixed" above. If NAAS is unable to fix the files, a red "failed" label will appear above. When NAAS has confirmed that the figure files meet our requirements, please download the file via the download option, and include these NAAS processed figure files when submitting your revised manuscript.

# Author comment: Author response to Decision Letter 1

**DOI:** 10.1371/journal.pgph.0006390.r002

18 Jun 2026

Attachment Submitted filename: Point-by-point response to the reviewers comments.pdf

# Peer review: Decision Letter 1

**DOI:** 10.1371/journal.pgph.0006390.r003

**Authors:** Matthew Berger

13 Jul 2026

PGPH-D-26-01253R1

Determinants of post-campaign measles vaccination coverage in Madagascar: A geographically stratified analysis

PLOS Global Public Health

Dear Dr. ALAIN,

Thank you for submitting your manuscript to PLOS Global Public Health. After careful consideration, we feel that it has merit but does not fully meet PLOS Global Public Health’s publication criteria as it currently stands. Therefore, we invite you to submit a revised version of the manuscript that addresses the points raised during the review process.

Please submit your revised manuscript by Aug 12 2026 11:59PM. If you will need more time than this to complete your revisions, please reply to this message or contact the journal office at globalpubhealth@plos.org. When you're ready to submit your revision, log on to https://www.editorialmanager.com/pgph/ and select the 'Submissions Needing Revision' folder to locate your manuscript file.

Please include the following items when submitting your revised manuscript:

- A letter that responds to each point raised by the editor and reviewer(s). You should upload this letter as a separate file labeled 'Response to Reviewers'.
- A marked-up copy of your manuscript that highlights changes made to the original version. You should upload this as a separate file labeled 'Revised Manuscript with Track Changes'.
- An unmarked version of your revised paper without tracked changes. You should upload this as a separate file labeled 'Manuscript'.

Guidelines for resubmitting your figure files are available below the reviewer comments at the end of this letter.

As the corresponding author, your ORCID iD is verified in the submission system and will appear in the published article. PLOS supports the use of ORCID, and we encourage all coauthors to register for an ORCID iD and use it as well. Please encourage your coauthors to verify their ORCID iD within the submission system before final acceptance, as unverified ORCID iDs will not appear in the published article. *Only*  the individual author can complete the verification step; PLOS staff *cannot*  verify ORCID iDs on behalf of authors.

We look forward to receiving your revised manuscript.

Kind regards,

Matthew N. Berger, PhD

Academic Editor

PLOS Global Public Health

**Journal Requirements:**

If the reviewer comments include a recommendation to cite specific previously published works, please review and evaluate these publications to determine whether they are relevant and should be cited. There is no requirement to cite these works unless the editor has indicated otherwise.

Please review your reference list to ensure that it is complete and correct. If you have cited papers that have been retracted, please include the rationale for doing so in the manuscript text, or remove these references and replace them with relevant current references. Any changes to the reference list should be mentioned in the rebuttal letter that accompanies your revised manuscript. If you need to cite a retracted article, indicate the article’s retracted status in the References list and also include a citation and full reference for the retraction notice.

**Additional Editor Comments (if provided):**

Academic Editor

There is still uncertainty around the qualitative approach in this study. You've stated that you followed a framework, but there is no reference to which one. If no published framework was followed, this must be included in the limitations section. Please check the previous report, as some comments made appear not to have been addressed, including the inclusion of reporting guidelines for qualitative research. In particular, consider using the Consolidated Criteria for Reporting Qualitative Research (COREQ) and providing the completed checklist as a supplementary file. Alternatively, the Standards for Reporting Qualitative Research (SRQR) may be appropriate. The previous comment on mixed methods does not appear to have been reviewed. Please refer to my last comment: It is unclear whether a formal mixed methods methodology was employed. As currently presented, the manuscript appears more consistent with a multi-method study, where quantitative and qualitative components are conducted alongside one another but without clear methodological integration. If the intention is to present this as a mixed methods study, I recommend explicitly describing the mixed methods design, rationale, and integration procedures. As reviewer 1 noted, I would also hope to see the integration of the quantitative and qualitative findings in the discussion, at a minimum. The following resources may be helpful: 1. Curry, L & Nunez-Smith, M 2016, Mixed methods in health sciences research : a practical primer 1st ed., SAGE, Los Angeles. 2. Poth, C. N. (Ed.). (2024). The Sage Handbook of Mixed Methods Research Design.

Reviewer 1

I am still of the view that the results of the qualitative components can be made more transparent and be better integrated into the discussion.

[Note: HTML markup is below. Please do not edit.]

Reviewers' comments:

Reviewer's Responses to Questions

**Comments to the Author**

1. If the authors have adequately addressed your comments raised in a previous round of review and you feel that this manuscript is now acceptable for publication, you may indicate that here to bypass the “Comments to the Author” section, enter your conflict of interest statement in the “Confidential to Editor” section, and submit your "Accept" recommendation.

Reviewer #1: All comments have been addressed

Reviewer #2: All comments have been addressed

**********

2. Does this manuscript meet PLOS Global Public Health’s publication criteria? Is the manuscript technically sound, and do the data support the conclusions? The manuscript must describe methodologically and ethically rigorous research with conclusions that are appropriately drawn based on the data presented.

Reviewer #1: Yes

Reviewer #2: Yes

**********

3. Has the statistical analysis been performed appropriately and rigorously?

Reviewer #1: Yes

Reviewer #2: Yes

**********

4. Have the authors made all data underlying the findings in their manuscript fully available (please refer to the Data Availability Statement at the start of the manuscript PDF file)?

The PLOS Data policy requires authors to make all data underlying the findings described in their manuscript fully available without restriction, with rare exception. The data should be provided as part of the manuscript or its supporting information, or deposited to a public repository. For example, in addition to summary statistics, the data points behind means, medians and variance measures should be available. If there are restrictions on publicly sharing data—e.g. participant privacy or use of data from a third party—those must be specified.

Reviewer #1: Yes

Reviewer #2: Yes

**********

5. Is the manuscript presented in an intelligible fashion and written in standard English?

PLOS Global Public Health does not copyedit accepted manuscripts, so the language in submitted articles must be clear, correct, and unambiguous. Any typographical or grammatical errors should be corrected at revision, so please note any specific errors here.

Reviewer #1: Yes

Reviewer #2: Yes

**********

6. Review Comments to the Author

Please use the space provided to explain your answers to the questions above. You may also include additional comments for the author, including concerns about dual publication, research ethics, or publication ethics. (Please upload your review as an attachment if it exceeds 20,000 characters)

**Reviewer #1:**  I am still of the view that the results of the qualitative components can be made more transparent and be better integrated into the discussion.

**Reviewer #2** : The authors have adequately addressed my previous comments and revised the manuscript accordingly. I have no further concerns and support the manuscript’s acceptance.

**********

7. PLOS authors have the option to publish the peer review history of their article (what does this mean?). If published, this will include your full peer review and any attached files.

**Do you want your identity to be public for this peer review?**  If you choose “no”, your identity will remain anonymous but your review may still be made public.

For information about this choice, including consent withdrawal, please see our Privacy Policy.

Reviewer #1: **Yes:**  Sunday charles Adeyemo

Reviewer #2: No

**********

[NOTE: If reviewer comments were submitted as an attachment file, they will be attached to this email and accessible via the submission site. Please log into your account, locate the manuscript record, and check for the action link "View Attachments". If this link does not appear, there are no attachment files.]

**Figure Resubmissions:**

While revising your submission, we strongly recommend that you use PLOS’s NAAS tool (https://ngplosjournals.pagemajik.ai/artanalysis) to test your figure files. NAAS can convert your figure files to the TIFF file type and meet basic requirements (such as print size, resolution), or provide you with a report on issues that do not meet our requirements and that NAAS cannot fix.

After uploading your figures to PLOS’s NAAS tool - https://ngplosjournals.pagemajik.ai/artanalysis, NAAS will process the files provided and display the results in the "Uploaded Files" section of the page as the processing is complete. If the uploaded figures meet our requirements (or NAAS is able to fix the files to meet our requirements), the figure will be marked as "fixed" above. If NAAS is unable to fix the files, a red "failed" label will appear above. When NAAS has confirmed that the figure files meet our requirements, please download the file via the download option, and include these NAAS processed figure files when submitting your revised manuscript.

# Author comment: Author response to Decision Letter 2

**DOI:** 10.1371/journal.pgph.0006390.r004

15 Jul 2026

Attachment Submitted filename: Point-by-point_response_to_the_reviewers_comments_auresp_2.pdf

# Editor report: Decision Letter 2

**DOI:** 10.1371/journal.pgph.0006390.r005

**Authors:** Matthew Berger

19 Jul 2026

PGPH-D-26-01253R2

Determinants of post-campaign measles vaccination coverage in Madagascar: A geographically stratified analysis

PLOS Global Public Health

Dear Dr. ALAIN,

Thank you for submitting your manuscript to PLOS Global Public Health. After careful consideration, we feel that it has merit but does not fully meet PLOS Global Public Health’s publication criteria as it currently stands. Therefore, we invite you to submit a revised version of the manuscript that addresses the points raised during the review process.

Please submit your revised manuscript by Aug 18 2026 11:59PM. If you will need more time than this to complete your revisions, please reply to this message or contact the journal office at globalpubhealth@plos.org. When you're ready to submit your revision, log on to https://www.editorialmanager.com/pgph/ and select the 'Submissions Needing Revision' folder to locate your manuscript file.

Please include the following items when submitting your revised manuscript:

- A letter that responds to each point raised by the editor and reviewer(s). You should upload this letter as a separate file labeled 'Response to Reviewers'.
- A marked-up copy of your manuscript that highlights changes made to the original version. You should upload this as a separate file labeled 'Revised Manuscript with Track Changes'.
- An unmarked version of your revised paper without tracked changes. You should upload this as a separate file labeled 'Manuscript'.

Guidelines for resubmitting your figure files are available below the reviewer comments at the end of this letter.

As the corresponding author, your ORCID iD is verified in the submission system and will appear in the published article. PLOS supports the use of ORCID, and we encourage all coauthors to register for an ORCID iD and use it as well. Please encourage your coauthors to verify their ORCID iD within the submission system before final acceptance, as unverified ORCID iDs will not appear in the published article. *Only*  the individual author can complete the verification step; PLOS staff *cannot*  verify ORCID iDs on behalf of authors.

We look forward to receiving your revised manuscript.

Kind regards,

Matthew N. Berger, PhD

Academic Editor

PLOS Global Public Health

**Journal Requirements:**

If the reviewer comments include a recommendation to cite specific previously published works, please review and evaluate these publications to determine whether they are relevant and should be cited. There is no requirement to cite these works unless the editor has indicated otherwise.

Please review your reference list to ensure that it is complete and correct. If you have cited papers that have been retracted, please include the rationale for doing so in the manuscript text, or remove these references and replace them with relevant current references. Any changes to the reference list should be mentioned in the rebuttal letter that accompanies your revised manuscript. If you need to cite a retracted article, indicate the article’s retracted status in the References list and also include a citation and full reference for the retraction notice.

**Additional Editor Comments (if provided):**

Thank you for submitting the revised version of the manuscript. I have a few remaining minor points for clarification.

Could you please review the references and ensure that the COREQ guideline is cited in the manuscript: Tong A, Sainsbury P, Craig J. Consolidated criteria for reporting qualitative research (COREQ): a 32-item checklist for interviews and focus groups. International Journal for Quality in Health Care. 2007;19(6):349–357. doi:10.1093/intqhc/mzm042.

The manuscript also refers to “(e.g., Ritchie & Spencer).” Please clarify whether the research team formally followed the Ritchie and Spencer framework during the analysis. Although some of the revised wording describing the analytical process appears consistent with elements of this framework, the reference currently seems to have been added retrospectively or somewhat forced into the Methods section.

If the framework was formally used, please remove “e.g.,” and include the appropriate reference within the manuscript. If it was not formally followed, please remove the reference and state in the limitations section that no formal analytical framework was used.

This clarification would help ensure that the methodological approach is reported accurately and transparently.

Thank you again for your revisions.

[Note: HTML markup is below. Please do not edit.]

**Reviewers' comments:**

[NOTE: If reviewer comments were submitted as an attachment file, they will be attached to this email and accessible via the submission site. Please log into your account, locate the manuscript record, and check for the action link "View Attachments". If this link does not appear, there are no attachment files.]

**Figure Resubmissions:**

While revising your submission, we strongly recommend that you use PLOS’s NAAS tool (https://ngplosjournals.pagemajik.ai/artanalysis) to test your figure files. NAAS can convert your figure files to the TIFF file type and meet basic requirements (such as print size, resolution), or provide you with a report on issues that do not meet our requirements and that NAAS cannot fix.

After uploading your figures to PLOS’s NAAS tool - https://ngplosjournals.pagemajik.ai/artanalysis, NAAS will process the files provided and display the results in the "Uploaded Files" section of the page as the processing is complete. If the uploaded figures meet our requirements (or NAAS is able to fix the files to meet our requirements), the figure will be marked as "fixed" above. If NAAS is unable to fix the files, a red "failed" label will appear above. When NAAS has confirmed that the figure files meet our requirements, please download the file via the download option, and include these NAAS processed figure files when submitting your revised manuscript.

# Author comment: Author response to Decision Letter 3

**DOI:** 10.1371/journal.pgph.0006390.r006

21 Jul 2026

Attachment Submitted filename: Point-by-point response to the reviewers comments V3.pdf

# Editor report: Decision Letter 3

**DOI:** 10.1371/journal.pgph.0006390.r007

**Authors:** Matthew Berger

30 Jul 2026

Determinants of post-campaign measles vaccination coverage in Madagascar: A geographically stratified analysis

PGPH-D-26-01253R3

Dear Dr ALAIN,

We are pleased to inform you that your manuscript 'Determinants of post-campaign measles vaccination coverage in Madagascar: A geographically stratified analysis' has been provisionally accepted for publication in PLOS Global Public Health.

Before your manuscript can be formally accepted you will need to complete some formatting changes, which you will receive in a follow up email. A member of our team will be in touch with a set of requests.

Please note that your manuscript will not be scheduled for publication until you have made the required changes, so a swift response is appreciated.

IMPORTANT: The editorial review process is now complete. PLOS will only permit corrections to spelling, formatting or significant scientific errors from this point onwards. Requests for major changes, or any which affect the scientific understanding of your work, will cause delays to the publication date of your manuscript.

If your institution or institutions have a press office, please notify them about your upcoming paper to help maximize its impact. If they'll be preparing press materials, please inform our press team as soon as possible -- no later than 48 hours after receiving the formal acceptance. Your manuscript will remain under strict press embargo until 2 pm Eastern Time on the date of publication. For more information, please contact globalpubhealth@plos.org.

Thank you again for supporting Open Access publishing; we are looking forward to publishing your work in PLOS Global Public Health.

Best regards,

Matthew N. Berger, PhD

Academic Editor

PLOS Global Public Health

***********************************************************

Reviewer Comments (if any, and for reference):
